# Dr. Herbert Stanley Greaves

**Published:** 1912-08-10

**Authors:** 


Dr. Herbert Stanley Greaves, of Bridgetown, Barbados, West Indies, has died at the early age of thirty-eight. He was until recently the senior resident surgeon at the General Hospital, Barbados. Dr. Greaves was a student at St. Bartholomew's Hospital, and qualified in 1899 at the Conjoint Board. He held the post of house physician at his own hospital, and was also house surgeon at the Hospital for Sick Children, Great Ormond Street.

